# The relationship between pollen monodiets and the activities of proteolytic systems in the fat body and hemolymph of honeybee workers

**DOI:** 10.1371/journal.pone.0326175

**Published:** 2025-06-18

**Authors:** Maciej Sylwester Bryś, Krzysztof Olszewski, Aneta Strachecka

**Affiliations:** 1 Department of Invertebrate Ecophysiology and Experimental Biology, Faculty of Environmental Biology, University of Life Sciences in Lublin, Lublin, Poland; 2 Subdepartment of Apidology, Institute of Biological Basis of Animal Production, Faculty of Animal Sciences and Bioeconomy, University of Life Sciences in Lublin, Lublin, Poland; Tanta University Faculty of Science, EGYPT

## Abstract

The homogenization of landscapes through the introduction of large-scale farms, the decline of biodiversity conditioned by high summer temperatures and dry weather, as well as the expansion of alien species determine the monodiet feeding of honeybees. In this study, we investigated the effect of monopollen feeding regimens (containing hazel, rapeseed, pine, buckwheat, Phacelia, and goldenrod) on the activity of the proteolytic system in the tergite 3, tergite 5 or sternite apian fat body, and hemolymph. We showed that pollen from rapeseed, Phacelia, buckwheat, and goldenrod increased the activities of acidic, neutral, and alkaline proteases and their inhibitors in the fat body and hemolymph when compared to the group fed with sugar candy only. The activities of proteases and their inhibitors in bees fed with pollen from hazel and pine were usually higher compared to the activities of honeybees fed with sugar candy only, but lower than in workers fed sugar candy with the pollen of entomophilous plants. Moreover, when comparing the proteolytic system activity between localizations/segments, the highest values were observed in tergite 5, regardless of what age the bees were and whether they were fed candy with added pollen. It is important to understand the impact of individual types of pollen in the context of potential future monodiets. Furthermore, the beneficial impact of Phacelia pollen to drive the rise of protease and protease inhibitor activities, helping to counteract negative environmental factors, can be supported by introducing, for example, flower mixtures for the insects or pollen-supplemented sugar candies for bees during periods without access to pollen.

## Introduction

In the 21st century, progressing industrialization, the development of technologies that generate electromagnetic fields [1,2], chemical contamination of the environment [3–6], the adulteration of bee products [7], frequent mowing of roadsides and railway lines [8], as well as the homogenization of agriculture through monoculture practices [9–13], among other things, have led to a massive decline in bee populations.

Pollen quality, defined as the content of essential and non-essential amino acids, plays a crucial role in the physiology of the honeybee [14,15]. It turns out that access to a large amount of pollen produced by a single plant (the so-called monodiet) may not ensure proper development of insects due to an unbalanced nutritional profile [16]. Firstly, humans, through large-area farm cultivation of, for example, rapeseed, buckwheat, sunflower, etc., expose bees to stress caused by an undifferentiated diet. Secondly, large-area crops of industrial cereals around the world, such as corn, rice, sorghum and soybeans, provide honey bee with poor quality pollen [17]. Moreover, cereal plants are wind-pollinated and do not produce nectar, which does not encourage bees to visit them. Thirdly, there is also the phenomenon of invasive alien species, such as the Canadian goldenrod (*Solidago canadensis* L.), which outcompetes native plant species and almost forces bees to use its floral resources [18,19]. Good practices to supplement nutritional gaps include sowing flower meadows and cultivating specific plants, such as Phacelia (*Phacelia tanacetifolia* Benth). Phacelia has a short growing season and produces large amounts of biochemically balanced pollen and nectar [20,21]. According to our previous research, Phacelia pollen increases the energy reserves stored in the fat body and increases the activities of oxidative stress markers, preventing adverse stressors [10,22]. The selection of pollen in our study was dictated by the basic diet of honeybees in Poland and Central Europe. Hazel (*Corylus* sp.) pollen is the first source of pollen, even before the flowering period of other insect-pollinated plants [23]. *Brassica napus* L. (hereinafter referred to as rapeseed) is a common oilseed crop cultivated in Canada, China, India, and across Europe. This plant produces pollen with a total protein content of 27% [24], making rapeseed a valuable food source for honeybees, bumblebees, and solitary bees. Pine (*Pinus sylvestris* L.) pollen is produced in large quantities, but it is not readily collected by foraging bees. Buckwheat (*Fagopyrum esculentum* Moench) blooms between July and early autumn and is a valuable source of pollen, although the biochemical composition of the pollen is poorly balanced. Goldenrod (*Solidago* sp.) is a common invasive plant, and in many countries this plant is the last source of food before wintering. Data concerning the amount of pollen produced as well as the total protein content percentage for the plants discussed in this manuscript are presented in Table 1.

**Table 1:** The amount of pollen produced and the percentage of total protein content.

Taxon	Pollen production	Total Protein Content [%]	References
*Corylus* sp.	0.17 kg/bush	no literature data available	[23]
*Brassica napus* L*.*	9.3 kg pollen per ha per day	27.27	[24]
*Pinus* sp.	100-1000 kg/ha	11.7-13.7	[25,26]
*Phacelia tanacetifolia* Benth	180 kg/ha	27.44	[20]
*Fagopyrum esculentum* Moench	25 kg/ha	9-11.4	[27]
*Solidago canadensis* L. *Solidago gigantea* Aiton	30.7 kg/ha 6.5 kg/ha	29.22 23.61	[28]

Hunger and malnutrition are serious problems facing the present-day world, affecting not only humans but also animals. Little is known about the impact of hunger and malnutrition on insect physiology [29]. As a result of anthropogenic threats related to climate change, honeybees are being increasingly exposed to nutritional stress [30,31]. Moreover, there is a link between nutritional stress and microbial infections of the insect digestive tract, which impair digestion and nutrient absorption, causing energy stress [32,33]. From a physiological perspective, a pollen-based diet conditions the development of hypopharyngeal glands, influencing jelly production, midgut histology, and the quantity and types of hemocytes in hemolymph. It also impacts hormone production, immunity peptide synthesis, gene expression, bee mass, energy metabolism and ultimately, longevity [10,11,22,34–39]. A properly balanced diet ensures homeostasis between the honeybee digestive tract, hemolymph, and fat body. Hemolymph serves as a reservoir of nutrients that are rapidly utilized, for example, during flight [40–42]. In the honeybee, the open circulatory system causes the fat body cells to be washed by the hemolymph, facilitating nutrient exchange. The fat body acts as the primary storage site for nutrients, such as protein, glycogen and triglycerides, derived from a pollen-based diet. Besides their storage function, fat body trophocytes secrete and detoxify organic substances [43–45]. The metabolic activity of oenocyte nuclei depends on the nutrients provided from a pollen diet [10]. Moreover, a pollen diet influences the synthesis of antimicrobial peptides (AMP) such as apidaecin, abaecin, defensin, and hymenoptaecin in the fat body [11]. AMP are short peptides that inhibit the viability of Gram-positive and Gram-negative bacteria, viruses and fungi [46,47].

Nutritional stress triggers the activation of the insect’s innate immune system. Honey bee innate immunity comprises cellular and humoral responses. Cellular immunity involves the action of hemocytes in the hemolymph, facilitating processes such as nodulation, encapsulation, and phagocytosis [48–50]. Humoral immunity involves the production of proteins that protect against pathogen invasion and development. This response includes the proteolytic system (proteases and protease inhibitors), the antioxidant system, and biochemical markers [49]. Proteolytic enzymes indirectly influence the activation of the phenoloxidase cascade [51]. The proteolytic system in honeybees consists of proteases (endo- and exoproteases) and protease inhibitors [52]. Proteases exhibit relative specificity depending on the pH. The activities of these enzymes are influenced by factors such as individual development, caste (queen, worker, drone), environmental pollution levels, physiological state, stress factors, pathogens like bacteria, viruses, fungi, exposure to substances, such as acaricides and biostimulants, and even the size of the comb cell as a factor within the bee environment [6,50,52–56]. Given the multitude of factors affecting proteases and their inhibitor activities, it is highly probable that the proteolytic system of honey bees is also shaped by pollen-based diets.

Therefore, we decided to investigate how the main pollens from Central and Eastern Europe and other regions, particularly in the context of a mono-diet, affect the proteolytic system of honeybees as one of their immunity mechanisms. We hypothesized that (Hypothesis 1) different types of pollen would affect proteolytic enzyme activities in the fat body and hemolymph in distinct ways, (Hypothesis 2) pollen from insect-pollinated plants would have a greater impact on increasing protease and protease inhibitor activities than pollen from wind-pollinated plants, and (Hypothesis 3) a diet based on sugar candy only may disturb the balance of proteases and their inhibitors in the fat body and hemolymph and be more harmful than a monodiet. The aim of the study was to determine the effects of pollen from wind-pollinated plants: hazel (*Corylus* sp.) and pine (*Pinus sylvestris* L.) and insect-pollinated ones: rapeseed (*Brassica napus* L.), Phacelia (*Phacelia tanacetifolia* Benth), buckwheat (*Fagopyrum esculentum* Moench), and goldenrod (*Solidago* sp.) on the activities of the proteolytic system in the tergite 3, tergite 5 or sternite fat bodies and in the hemolymph.

## Materials and methods

### Pollen loads collection and microscopic pollen analysis

Monofloral pollen diets were chosen to reflect the most frequently occurring plant pollen types in Central and Eastern Europe and other parts of the world. Fresh pollen loads were collected from hives using pollen traps at various times throughout the flowering season, spanning the period from May 2022 to October 2023. The colonies were subject only to minimal disturbance due to pollen collection, and all other experimental conditions were carefully controlled to ensure their undisturbed development. The areas in Poland from which the pollen loads were sourced are considered unpolluted and free from heavy metal and pesticide contamination. A manual colour sorting process was implemented to isolate the dominant pollen type from each pollen load. Microscopic preparations were then made and analyzed using the methods described by Filipiak et al. [16] to confirm the botanical origin of the pollen. In this way, pollens were obtained from wind-pollinated plants: hazel (*Corylus* sp.) and pine (*Pinus sylvestris* L) and insect-pollinated ones: rapeseed (*Brassica napus* L.), Phacelia (*Phacelia tanacetifolia* Benth), buckwheat (*Fagopyrum esculentum* Moench), and goldenrod (*Solidago* sp.). The percentage of the dominant pollen in the sample of pollen loads was at least 80%. The sorted pollen was maintained at a temperature of −25°C until it was utilized for the preparation of sugar candy.

### Experimental design

**Fig 1:**
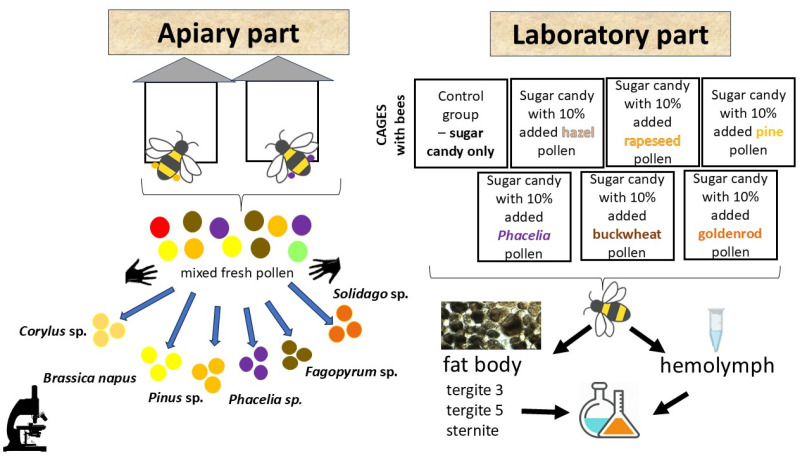
Schematic overview of the experiment design.

Studies were conducted on bees from the apiary of the University of Life Sciences in Lublin, Poland (51.224039N–22.634649E), originating from colonies of similar strength and age, which were not subjected to any treatment with antibiotics or fungicides. In this way, it was assumed that the bees’ immune system functioned naturally, without the influence of harmful external factors, as well as without the action of stimulants. The experiment began with obtaining 1-day-old worker honeybees (*Apis* *mellifera carnica*) according to the method of Bryś et al. (2025) [22]. Freshly emerged worker bees were placed in 12 cages per group (40 workers in each cage), with a division into 7 groups: a control group fed with sugar candy and six groups fed from the first day with sugar candy with the addition of 10% of the given pollen: hazel, rapeseed, pine, Phacelia, buckwheat, and goldenrod (Fig 1). We did not record any spores of *Vairimorpha*/*Nosema* in the cage test bees during the experiment (each group of sample worker bees was checked when 7 days old and 14 days old). The optimal conditions were maintained in a climate-controlled chamber with a constant temperature of 35°C and 65% relative humidity. Every two days, dead workers were removed from the cages and food and water were supplemented ad libitum. When the workers were 7 and 14 days old, 2 of them were collected from each cage (n = 24 workers per group, with the exception of the group fed rapeseed, where n = 12 workers were sampled) and laboratory analyzes were performed. Due to frequent gut rupture during manual removal of the intestines in bees fed with rapeseed pollen, which led to contamination of the fat body, only 12 workers were included in the analysis. This produced the following dataset: 24 one-day-old workers + (24 worker bees × 7 feeding groups) × 2 age groups (7- and 14-day-old).

No specific permissions were required for access to the field site, as the apiary is owned by the University of Life Sciences in Lublin. Moreover, the experiment was conducted under controlled laboratory conditions.

### Hemolymph, fat body collection and determination of proteolytic system activities

Hemolymph samples were collected from living bees using a glass capillary (20 μl; ‘end to end’ type; without an anticoagulant; Medlab Products, Raszyn, Poland) according to the Łoś and Strachecka’s [57] method into a sterile Eppendorf tube containing 200 μL of 0.6% *natrium chloratum* (concentration dedicated for honey bees) and immediately frozen at −23°C. Using a stereomicroscope, the fat body from tergite 3, tergite 5 and sternite was prepared [45]. The proteolytic system activity was determined in fat body homogenates and hemolymph samples:

- activities of acidic (pH 2.4), neutral (pH 7.0) and alkaline (pH 11.2) proteases according to the Anson method [58] modified by Strachecka et al. [52]
- activities of natural inhibitors of acidic (pH 2.4), neutral (pH 7.0) and alkaline (pH 11.2) proteases according to the Lee and Lin method [59].

The supernatants were analyzed spectrophotometrically (Synergy HTX (S1LFA); Warsaw, Poland) to measure absorbance at 280 nm.

### Statistics

Statistical analyses were performed using Statistica formulas (TIBCO Software, Palo Alto, CA, USA), 13.3 (2017) version for Windows — StatSoft Inc., Tulsa, OK, USA. Data distribution was checked using the Shapiro-Wilk test. Proteolytic enzyme activities in different tissues/ fat body locations (tergite 3, tergite 5, sternite and hemolymph) in the 1-day-old workers (n = 24 bees) were compared using the Mann-Whitney U test. For each type of pollen, the effect of tissue/ fat body locations (tergite 3, tergite 5, sternite and hemolymph) on the activities of acidic, neutral and alkaline proteases and their inhibitors was assessed in the 7- and 14-day-old workers (n = 24 bees per group, with the exception of the group receiving sugar candy with rapeseed where n = 12). Normally distributed data were analysed with ANOVA and the Kruskal–Wallis test was employed for non-normally distributed data. Proteolytic enzymes activities for hazel, rapeseed, pine, Phacelia, buckwheat and goldenrod pollen were compared inside the tissue/location of fat body (fat body from tergite 3, tergite 5, sternite) and hemolymph. A comparison was made with a control group within the age group for the 7- and 14-day-old insects, respectively. The Student t test was used for normally distributed data and the Mann-Whitney U test was employed for non-normally distributed data. According to the above model, a comparison was also made between the influence of pollen from wind-pollinated plants (hazel and pine) and that from insect-pollinated ones (rapeseed, Phacelia, buckwheat, goldenrod) on the enzyme activities.

## Results

In the one-day-old worker bees, the tissue location (hemolymph or fat body from tergite 3, tergite 5 or sternite) had a statistically significant effect on the activities of proteases and their inhibitors (Table 2). Regardless of the tissue type and fat body location, the highest enzymatic activities were recorded for alkaline proteases. The statistically significant highest activities of proteases were identified in the hemolymph and the lowest in tergite 3 and sternite of 1-day-old honeybee workers (p ≤ 0.01). The similar trends were observed for protease inhibitors. The activities of protease inhibitors in the 1-day-old workers were statistically significant in the comparison between the hemolymph and different fat body locations at p ≤ 0.05 (Table 2). Regardless of the tissue type and fat body location (tergite3, tergite 5 and sternite), the highest inhibitor activities were observed at acidic pH. At both 7 and 14 days of age, in each of the feeding groups, the tissue/ fat body locations (tergite 3, tergite 5, sternite fat body or hemolymph) had a statistically significant effect on the activities of proteases and their inhibitors (Table 3).

**Table 2:** Activities of the proteolytic system in the hemolymph and fat body (tergite 3, tergite 5 or sternite) of 1-day-old *A. mellifera* L. workers.

	Protease Activities (U/mg)			Protease Inhibitor Activities (U/mg)		
Tissue/location	Acidic	Neutral	Alkaline	Acidic	Neutral	Alkaline
Hemolymph	0.13 (±0.02) ^A^	0.19 (±0.02) ^A^	2.32 (±0.29) ^A^	2.91 (±0.37) ^A^	0.58 (±0.07) ^A^	1.12 (±0.14) ^A^
Tergite 3	0.07 (±0.01) ^BD^	0.11 (±0.01) ^B^	1.24 (±0.08) ^Bd^	1.53 (±0.10) ^BDa^	0.31 (±0.02) ^BD^	0.61(±0.04) ^BD^
Tergite 5	0.08 (±0.01) ^BC^	0.12 (±0.01) ^C^	1.45 (±0.05) ^BC^	1.79 (±0.07) ^BC^	0.31 (±0.02) ^BC^	0.61 (±0.03) ^BC^
Sternite	0.07 (±0.01) ^BD^	0.10 (±0.01) ^D^	1.21 (±0.02) ^BD^	1.64 (±0.02) ^BDb^	0.34 (±0.01) ^BD^	0.79 (±0.01) ^BD^

The average protease activities and their inhibitors in a bee organism. Standard deviation is shown in round brackets. Different uppercase letters A, B, C, D indicate statistical differences between the tissue/ fat body location (in columns) at the p ≤ 0.01 significance level; different lowercase letters a and b at the p ≤ 0.05 significance level.

**Table 3:** Effect of tissue (fat body, hemolymph)/ fat body location (tergite 3, tergite 5, sternite) in the different age groups of workers (7 and 14 days) on the activities of proteases and their inhibitors in the *A*. *mellifera* workers fed sugar candy only (control group) and in those receiving sugar candy with a pollen addition.

Groups	Age of workers											
	7-day-old						14-day-old					
	Acidic proteases	Neutral proteases	Alkaline proteases	Acidic proteases inhibitors	Neutral proteases inhibitors	Alkaline proteases inhibitors	Acidic proteases	Neutral proteases	Alkaline proteases	Acidic protease inhibitors	Neutral protease inhibitors	Alkaline protease inhibitors
Control gr.	H = 89.07 p = 0.000	F = 89.07 p = 0.000	F = 89.07 p = 0.000	F = 80.23 p = 0.000	F = 75.52 p = 0.000	F = 80.18 p = 0.000	H = 89.07 p = 0.000	H = 89.07 p = 0.000	H = 89.07 p = 0.000	H = 89.07 p = 0.000	H = 89.07 p = 0.000	H = 89.07 p = 0.000
Hazel	H = 89.72 p = 0.000	H = 89.72 p = 0.000	H = 89.72 p = 0.000	H = 86.51 p = 0.000	H = 89.72 p = 0.000	H = 65.90 p = 0.000	H = 89.08 p = 0.000	H = 89.08 p = 0.000	H = 89.08 p = 0.000	H = 89.08 p = 0.000	H = 89.08 p = 0.000	H = 89.08 p = 0.000
Rapeseed	H = 44.08 p = 0.000	H = 44.08 p = 0.000	H = 44.08 p = 0.000	H = 44.08 p = 0.000	H = 44.08 p = 0.000	H = 43.61 p = 0.000	H = 44.08 p = 0.000	H = 44.09 p = 0.000	H = 44.09 p = 0.000	H = 44.09 p = 0.000	H = 44.09 p = 0.000	H = 44.09 p = 0.000
Pine	H = 89.07 p = 0.000	H = 89.07 p = 0.000	H = 89.07 p = 0.000	F = 88.95 p = 0.000	F = 89.07 p = 0.000	H = 87.09 p = 0.000	H = 89.08 p = 0.000	H = 89.08 p = 0.000	H = 89.08 p = 0.000	H = 89.08 p = 0.000	H = 89.08 p = 0.000	H = 89.08 p = 0.000
Phacelia	H = 88.13 p = 0.000	H = 88.13 p = 0.000	H = 88.13 p = 0.000	H = 88.14 p = 0.000	H = 88.14 p = 0.000	H = 81.96 p = 0.000	H = 87.17 p = 0.000	H = 87.16 p = 0.000	H = 87.17 p = 0.000	H = 87.18 p = 0.000	H = 87.18 p = 0.000	H = 87.18 p = 0.000
Buckwheat	H = 89.07 p = 0.000	H = 89.07 p = 0.000	H = 89.07 p = 0.000	H = 89.07 p = 0.000	H = 89.07 p = 0.013	H = 88.05 p = 0.000	H = 89.08 p = 0.000	H = 89.08 p = 0.000	H = 89.08 p = 0.000	H = 89.08 p = 0.000	H = 89.08 p = 0.000	H = 89.08 p = 0.000
Goldenrod	H = 89.06 p = 0.000	H = 89.09 p = 0.000	H = 89.08 p = 0.000	F = 84.95 p = 0.000	F = 89.08 p = 0.000	H = 86.88 p = 0.000	H = 89.07 p = 0.000	H = 89.07 p = 0.000	H = 89.08 p = 0.000	H = 89.08 p = 0.000	H = 89.07 p = 0.000	H = 89.07 p = 0.000

H – statistical value in the Kruskal–Wallis test; F – value of Fisher’s test in ANOVA; p – probability value.

**Fig 2:**
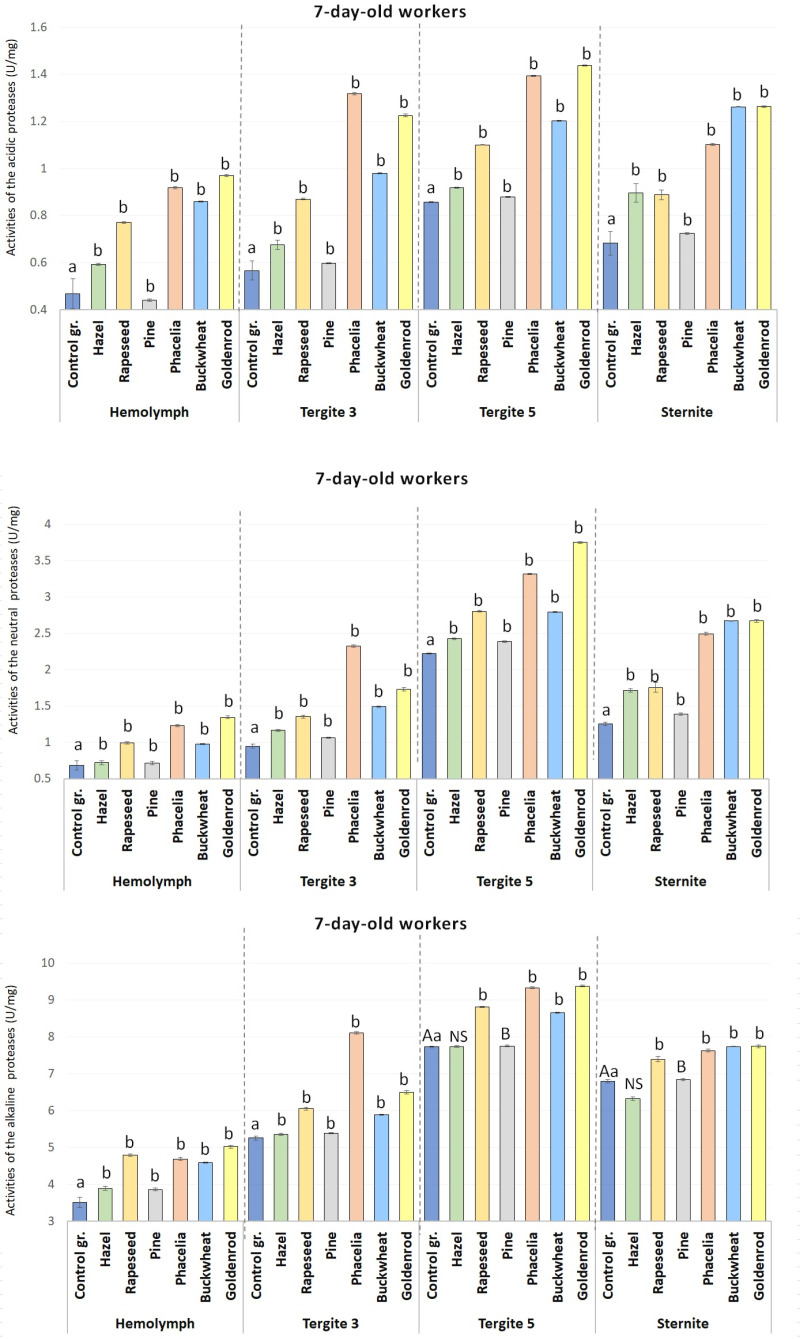
The mean activities of acidic, neutral, and alkaline proteases in the hemolymph and in the tergite 3, tergite 5 and sternite fat body in the 7-day-old workers fed sugar candy only and in those receiving sugar candy with various pollen additions; a, b – discrepancies between the workers administered pollen and the control ones in identical tissues/sites are meaningful at p ≤ 0.05; A, B – discrepancies between the workers administered pollen and the control ones in identical tissues/sites are meaningful at p ≤ 0.01; n = 24 (with the exception of the group fed rapeseed where n = 12); NS – not significant; vertical bars indicate standard deviation.

**Fig 3:**
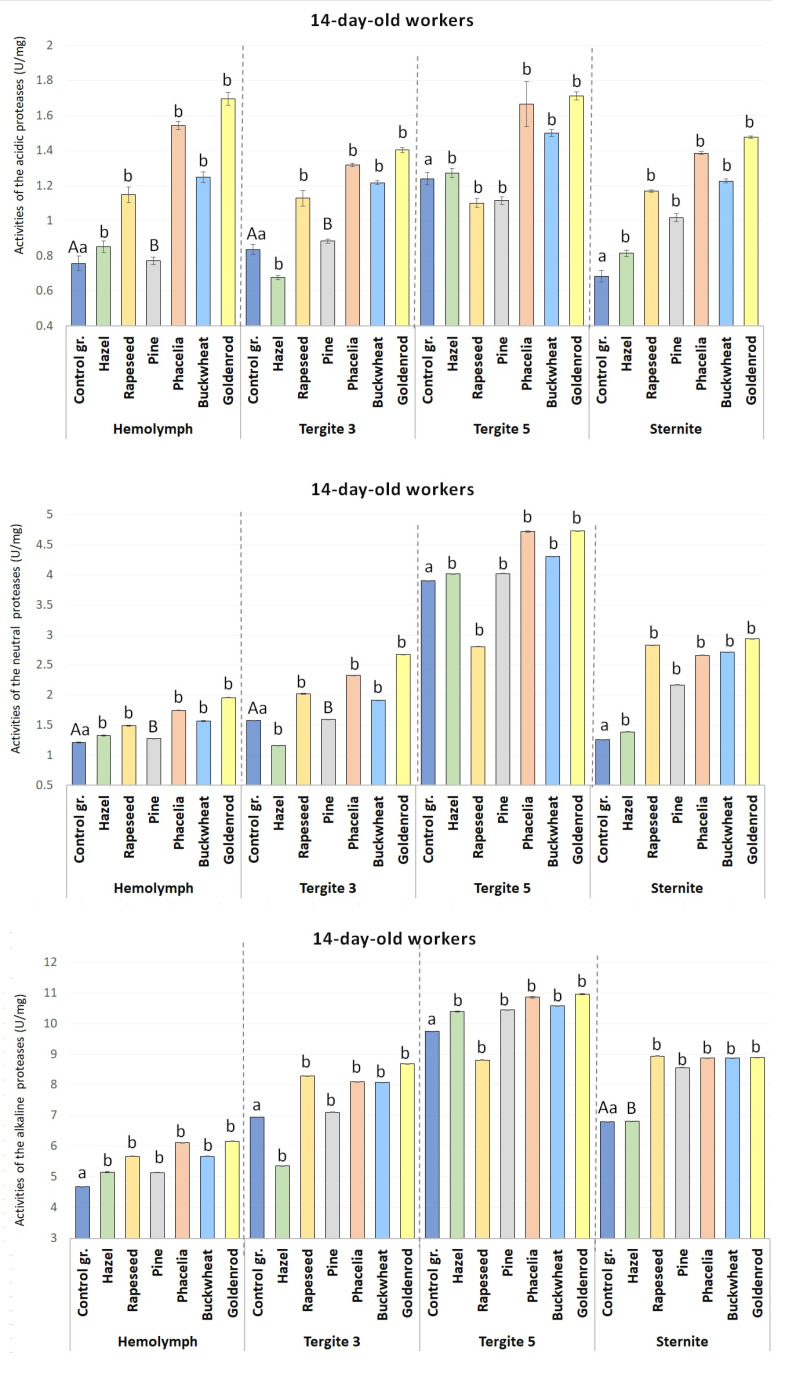
The mean activities of acidic, neutral, and alkaline proteases in the hemolymph and the fat body from tergite 3, tergite 5 and sternite in the 14-day-old workers fed sugar candy only and in those receiving sugar candy with various pollen additions; a, b – discrepancies between the workers administered pollen and the control ones in identical tissues/sites are meaningful at p ≤ 0.05; A, B – discrepancies between the workers administered pollen and the control ones in identical tissues/sites are meaningful at p ≤ 0.01; n = 24 (except for the group fed rapeseed where n = 12); vertical bars indicate standard deviation.

Except for the acid protease activities in tergite 5 of the rapeseed-fed 14-day-old workers, protease activities in the hemolymph and the tergite 3, tergite 5, and the sternite apian fat bodies were statistically significant (p ≤ 0.01) in the 7- and 14-day-old workers consuming rapeseed, Phacelia, buckwheat, and goldenrod compared to those fed sugar candy only (Figs 2 and 3).

Protease activities in the hemolymph increased with the age of the (1-, 7- and 14-day-old) workers on all the pollen monodiets and in the control group (Figs 2 and 3).

As compared to the control group, the acid, neutral and alkaline protease activities were statistically significantly higher in the 7-day-old (except pine in hemolymph for acid protease) and 14-day-old workers fed with a 10% pollen supplement (except for alkaline protease in tergite 5 and sternite in the 7-day-old-workers) (Figs 2 and 3).

**Fig 4:**
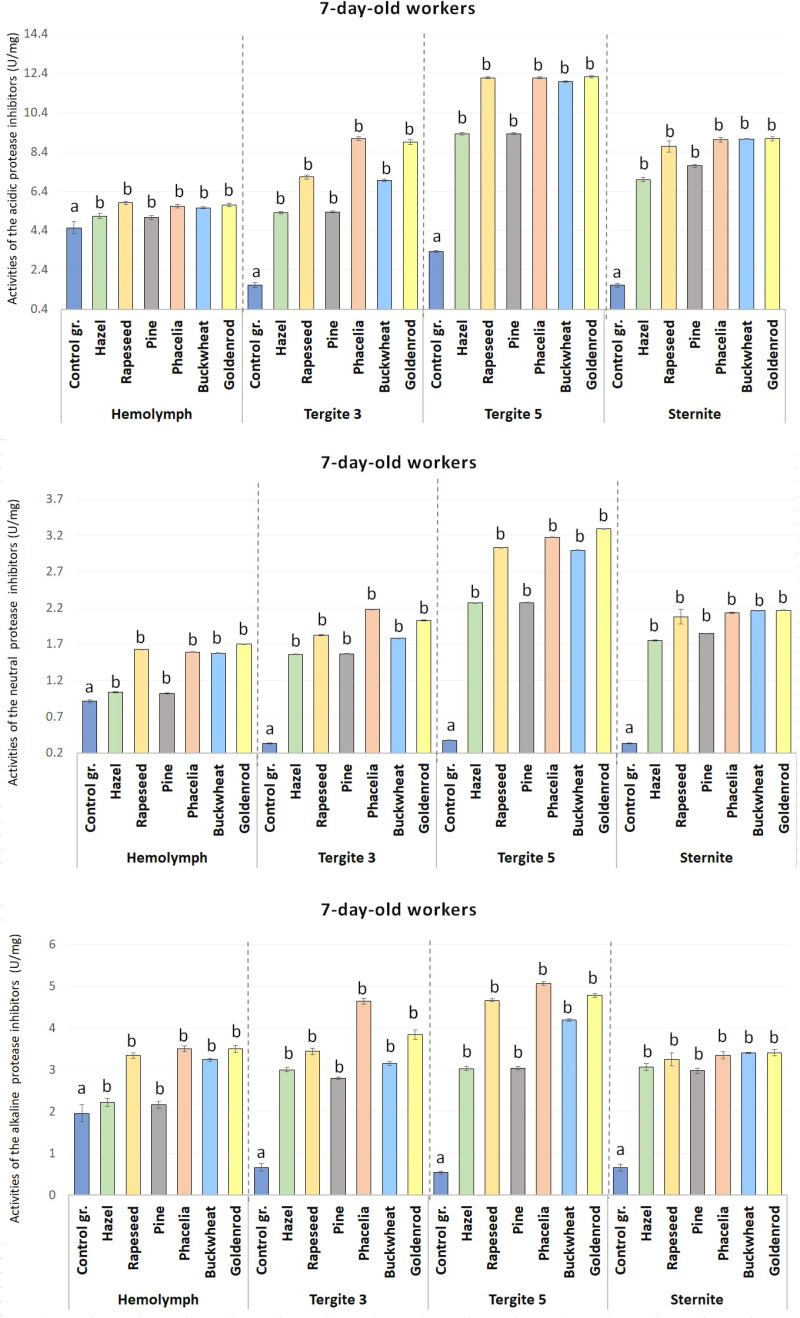
The mean activities of acidic, neutral, and alkaline protease inhibitors in the hemolymph and the tergite 3, tergite 5 and sternite fat bodies in the 7-day-old workers fed sugar candy only and in those receiving sugar candy with various pollen additions; a, b – discrepancies between the workers administered pollen and the control ones in identical tissues/sites are meaningful at p ≤ 0.01; n = 24 (with the exception of the group fed rapeseed where n = 12); vertical bars indicate standard deviation.

**Fig 5:**
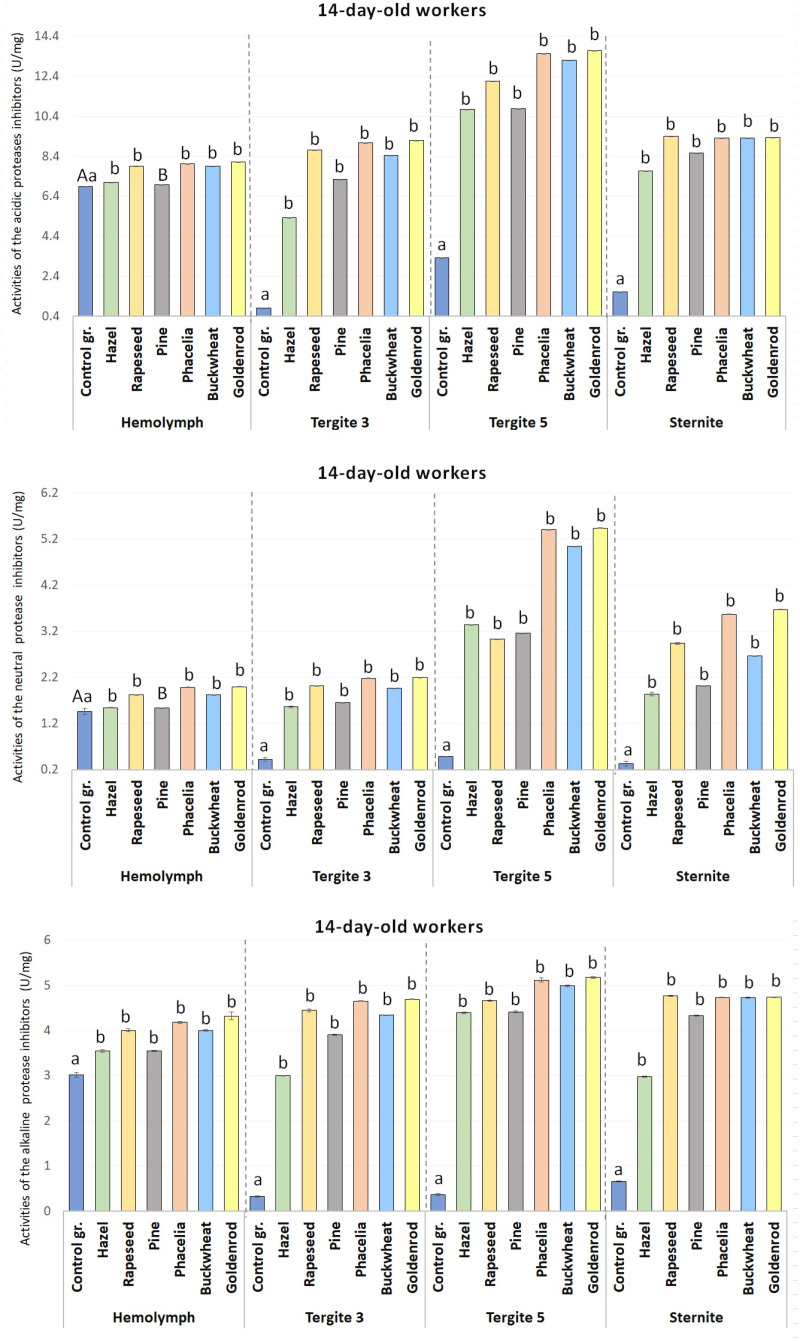
The mean activities of acidic, neutral, and alkaline protease inhibitors in the hemolymph and the tergite 3, tergite 5 and sternite fat bodies in the 7-day-old workers fed sugar candy only and in those receiving sugar candy with various pollen additions; a, b – discrepancies between the workers administered pollen and the control ones in identical tissues/sites are meaningful at p ≤ 0.05; A, B – discrepancies between the workers administered pollen and the control ones in identical tissues/sites are meaningful at p ≤ 0.01; n = 24 (except for the group fed rapeseed where n = 12); vertical bars indicate standard deviation.

The activities of acidic, neutral and alkaline protease inhibitors in the hemolymph increased with age regardless of the group (Table 2, Figs 4 and 5). In the tergite 3, tergite 5, and sternite fat bodies and also in the hemolymph, the activities of acid and alkaline protease inhibitors in the bees fed sugar candy only (control group) at 7 and 14 days of age were significantly lower as compared to all the pollen monodiets (p ≤ 0.01) (Fig 5) even in those receiving hazel pollen or pine pollen candy (p ≤ 0.05) (Figs 4 and 5).

No significant differences between the bees fed hazel and pine pollen were found for the activities of proteases or protease inhibitors in the hemolymph between the 7- and 14-day-old workers (p > 0.05). Hovewer, the enzymatic activities of both proteases and their inhibitors in the hemolymph of 7- and 14-day-old workers fed with pollen from wind-pollinated plants were consistently lower than those recorded for bees fed with pollen from insect-pollinated plants. Proteolytic activities in the fat body (tergite 3, tergite 5 and sternite) of the 7- and 14-day-old workers fed with pollen produced by insect-pollinated plants (rapeseed, Phacelia, buckwheat and goldenrod) were significantly higher than those determined for the bees fed with candy containing hazel or pine pollen (p = 0.01).

## Discussion

It is widely recognized that the expression of the immune system in vertebrates and invertebrates, including pollinating insects, depends closely on their nutritional status [60–65]. In an era of anthropopressure, the growth of large-scale farms and monocultures, bee dietetics is a rapidly developing field [66]. Most publications focus on the quality of pollen (its biochemical composition) [60,67], omitting aspects related to the impact of this food on the physiology and immunity of honeybees. Therefore, our publication fits into this scientific discourse and complements the available knowledge with these aspects. In our study, we utilized pollens from plants commonly consumed by bees throughout Europe, including hazel, rapeseed, pine, Phacelia, buckwheat, and goldenrod.

We have shown that the activity of the proteolytic system in bees fed with one type of pollen is higher than in those fed with sugar candy only (Hypothesis 3). This leads to the conclusion that although a monodiet is a stress factor for bees, in comparison to feeding bees with only sugar, its influence on the physiology of the bee organism can be considered beneficial. As indicated by [68], in order to build the protein potential of a bee organism, pollen cell walls are dismantled in the digestive tract by various mechanisms (e.g., enzymes or osmotic shock) to get to the nutrient-rich cytoplasm. These compounds enter the fat body through the hemolymph, where they participate in the synthesis of proteins, including those that are part of the proteolytic system.

The differences in the activities of the proteolytic system compounds in workers fed with different types of pollen may result firstly from different concentrations of total proteins in each of the analyzed pollens, cf. Table 1. Secondly, the compounds can be digested and absorbed into the organism in different degrees [69–70]. It is worth adding that the contents of proteins, carbohydrates, lipids and other biological compounds in pollen are determined by various geographical factors (plant origin and environmental conditions) [71], and the question arises whether the differences in the biochemical composition of pollen within the same species are significant enough to affect the proteolytic system of bees. This issue requires clarification in further studies. Basulado et al. [72] also noted that the type of pollen consumed influenced the total protein concentrations in the fat body of bees.

Moreover, Alaux et al. [35] showed a correlation between the crude protein contents of pollens and insect immunity. Also, Danihlik et al. [11] and Negri et al. [62] showed that, by providing exogenous amino acids, bee pollen affects peptide synthesis and the regulation of immune gene expression. As our studies have shown, the activities of proteases and their inhibitors depended on the type of pollen and were always higher in the bees fed with candy supplemented with rapeseed, Phacelia, buckwheat and goldenrod pollen as compared to those fed with hazel and pine candy (Hypotheses 1 and 2). In the case of pine pollen, the activity of this system were in many cases lower than in bees fed with sugar candy alone. Although the activity of the proteolytic system was the lowest in the tissues of bees fed sugar candy with hazel pollen compared to the other pollens, it was still higher than in the control group. These results are consistent with our previous observations [10], in which we showed that the protein concentrations in the hemolymph and fat body are higher after bees have consumed food supplemented with pollen from entomophilous plants rather than from anemophilous plants. The wind-pollinated plants produce pollen that is low in crude protein. In many areas of Central and Eastern Europe, hazel is the primary pollen source in early spring, and honey bees are almost entirely reliant on it.

The results of this article clearly confirm that hazel and pine pollen are very good food for bees after winter or during periods without nectar production and provide them with a large dose of sugars necessary for energy production to rear brood [10] and also for metabolic processes related to cellular and humoral immune responses [58,60]. This is important at a time of climate change, which results in accelerated flowering of plants, modifications in the availability of floral resources [73,74] and temperature changes, especially in autumn and winter [75]. Therefore, diets based on late summer and early autumn pollens, such as Phacelia, buckwheat and goldenrod, by moderating the activity of the proteolytic system, consequently influence the vitality, longevity and successful overwintering of bees [76]. Particularly high activities of proteases and protease inhibitors were observed in the bees fed with Phacelia pollen candy. Beekeepers frequently sow Phacelia in the summer (between June and July/August) to provide bees with a consistent supply of pollen and nectar. The rapid growth and flowering of this plant make it ideal for agricultural areas, offering protein-rich pollen, especially when bee food sources are scarce. Concerning proteolytic activity, goldenrod pollen showed equally high, although statistically different, values compared to rapeseed, buckwheat and Phacelia pollen. It should be noted that there are different species (including invasive species) of goldenrod, and their biochemical composition may differ [18,19,28]. These differences could potentially influence the activity of proteases and their inhibitors in the hemolymph and fat body of the honeybee. Therefore, further studies are needed to assess whether different goldenrod species exert distinct effects on these enzymatic activities.

**Fig 6:**
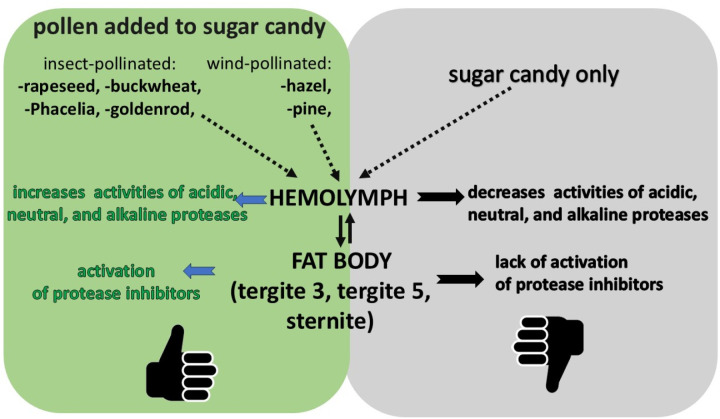
The effect of the pollen monodiets on protease and protease inhibitor activities in the *A. mellifera* workers fed sugar candy only (control group) and in those receiving sugar candy with a pollen addition.

We have shown that the activity of the proteolytic system increased with the age of the workers, both in the hemolymph and in the fat body. On the first day of life, higher activities of these compounds were observed in the bee hemolymph as compared to the different fat body locations. However, on the following days, the opposite is usually the case. It can therefore be assumed that a newly emerged bee has reserves of active proteases and their inhibitors synthesized during the preimaginal period. In order for this system to perform its function efficiently, it requires the additional synthesis and activation of individual proteases and protease inhibitors, for which protein from pollen is necessary (Fig 6). It is worth noting here that the activity of the proteolytic system has so far been described only in the hemolymph and no relationship has been demonstrated between this tissue and the fat body, especially using the segmental approach, in older bees. Performing studies of only 1-day-old bees, Strachecka et al. [50] observed that the activities of these compounds were the highest in the fat body of tergite 3. The differences in the results between these two experiments may stem from the season in which they were conducted, the conditions that prevailed in the colonies, and other factors (e.g., stressors). Strachecka et al. [52] do not provide any information about the health condition of the bees in their publication. The bees for our experiment came from strong and healthy colonies. Undoubtedly, an innovative aspect of the present study is to determine the effect of the particular types of pollen on the activity of the proteolytic system in different tissues (hemolymph *vs*. fat body) and different fat body locations (tergite 3 *vs*. tergite 5 *vs*. sternite). When comparing the activities of the proteolytic system between the respective fat body locations/segments, we observed the highest values in tergite 5, regardless of the age of the bees fed with pollen-supplemented candy. In line with Bryś et al.‘s [22] studies, this suggests that tergite 5 performs detoxification and neutralization functions, and these two (proteolytic and antioxidant) systems cooperate to eliminate the negative effects of active threats (e.g., pathogens, pesticides, etc.). Proteolytic enzymes “cut” the pathogen’s proteins into smaller units [53]. Increased activities of protease inhibitors provide protection for the bee organism against proteases produced by the pathogens, preventing them from penetrating body cavities and developing [51]. Strachecka et al. [52] demonstrated that acidic protease inhibitors target pathogenic fungi, basic inhibitors target bacteria and viruses, and neutral inhibitors target other stressors. Thus, a diet solely based on sugar candy, without access to protein, makes bees more susceptible to diseases and parasities. The associated reactions generate reactive oxygen species, which are removed by antioxidants [77]. Moreover, intercellular proteolytic enzymes can recognize and preferentially degrade oxidatively damaged proteins to amino acids [52]. The differentiation of the activities of proteases and their inhibitors in different fat body locations/segments, thus, corroborates the segmented organization of the subcuticular fat body, as proposed by Strachecka et al. [44]. The segmented structure of the fat body underlies the varying activities of proteolytic and antioxidant enzymes (particularly in tergite 5) and energy compounds (in tergite 3 and the sternite), implying distinct physiological functions of the individual fat body segments [10,22,52]. Moreover, higher activities of proteases and their inhibitors generated by a pollen diet will constitute a kind of barrier against other harmful factors such as *Vairimorpha*/*Nosema*, *Varroa*, etc. Interestingly, if the bees did not have access to pollen (a protein-free diet; control group), then it was not in the fat body but in the hemolymph of the 7- and 14-day-old workers that the highest activities of proteases and protease inhibitors were identified, just like in 1-day-old bees. We can therefore conclude that the lack of pollen in the diet limits the synthesis and activation of the compounds of the proteolytic system. This observation clearly verifies our hypothesis that a diet based on sugar candy only may disturb the proteolytic system activity and pose a potential risk of exposure to other stressors.

Various stressors, such as *Varroa destructor*, pesticides like imidacloprid, electromagnetic fields, and beekeeping practices (e.g., the comb cell size, formic acid use) affect the proteolytic system [6,54,56,78]. For example, exposing bees to a 50 Hz electric field at different intensities for 12 hours increased protease activities [78]. Paleolog et al. [6] showed that imidacloprid destabilizes the hemolymph proteolytic system of bees and impairs the cuticular proteolytic layer. In our study, the monodiet, considered an environmental stressor, did not significantly stress the hemolymph proteolytic system but performed better than a sugar-only diet. To counteract biotic, abiotic, and anthropogenic stressors, natural biostimulants such as caffeine, coenzyme Q10, curcumin, and the hemp extract are recommended, as they enhance protease and protease inhibitor activities in honeybee hemolymph [50,55,79,80]. However, although biostimulants have a positive effect, we believe that pollen-based diets are more desirable for stimulating the proteolytic system, as they provide protection against other stressors. We confirmed that a balanced diet is essential for the proper functioning of the immune system. Hence, further studies are needed to establish the relationship between nutrition and immunity in the face of other stressors.

## Conclusion

Large-scale cultivation of oilseed plants, multi-hectare cereal crops, and the expansion of invasive plants lead to honeybees subsisting on monodiets. That is why our research fits into the current trend and scientific discourse. Understanding the associated biochemical mechanisms, also in terms of the immune system, is the starting point for attempting to stop the decline in the numbers of pollinating insects. To our knowledge, our research is the first to show the effect of the main pollen-producing plants (hazel, rapeseed, pine, Phacelia, buckwheat and goldenrod) that constitute the basic source of pollen from spring to autumn in many European countries on the immune system, in relation to the hemolymph and the segmental structure of the subcuticular fat body in *A. mellifera*.

We have proven that the activity of the proteolytic system in bees fed with one type of pollen is higher than in those receiving sugar candy only. Although a monodiet is a stress factor for bees, its effect on the physiology of a bee’s body can be considered beneficial in comparison with feeding the insects exclusively with sugar candy. The activities of proteases and their inhibitors depend on the type of pollen and were always higher in the bees fed with candy supplemented with rapeseed, Phacelia, buckwheat and goldenrod pollen (insect-pollinated plants) compared to those fed with hazel and pine (wind-pollinated plants). It can therefore be concluded that pollens of wind-pollinated plants are a valuable supplement to the food base also in periods without nectar.

The highest activities of proteases and protease inhibitors were observed in the bees fed with candy enriched with Phacelia pollen. This is an indication for beekeepers and farmers to increase the areas of this plant. In practical terms for beekeepers, a 10% addition of pollen to sugar candy will be a viable and economical solution during the spring development or periodic gaps in the availability of pollen sources. A multi-pollen diet is always more beneficial for the bee’s body than a mono-pollen diet, but in order to understand the mechanisms in action here, the feeding regimen should be considered in the context of a monodiet. Notably, goldenrod pollen showed proteolytic activity comparable to that of other valuable insect-pollinated plants such as rapeseed and buckwheat.

The fat body of tergite 5 is the place where proteases and inhibitors activities are the highest compared to other locations of this tissue (sternite, tergite 3) and hemolymph. This may indicate that it is in the fat body of tergite 5 that the mechanisms related to detoxification and neutralization function, and this system together with antioxidant activities cooperate to eliminate the negative effects of threats (e.g., pathogens, pesticides, etc.).
